# Simplified molecular detection of *Leishmania *parasites in various clinical samples from patients with leishmaniasis

**DOI:** 10.1186/1756-3305-3-13

**Published:** 2010-03-02

**Authors:** Claire M Mugasa, Thierry Laurent, Gerard J Schoone, Frank L Basiye, Alfarazdeg A Saad, Sayda el Safi, Piet A Kager, Henk DFH Schallig

**Affiliations:** 1 Department of Veterinary Parasitology and Microbiology, Faculty of Veterinary Medicine, Makerere University Kampala, Kampala, Uganda; 2 Koninklijk Instituut voor de Tropen (KIT)/Royal Tropical Institute, KIT Biomedical Research, Amsterdam, the Netherlands; 3 Coris BioConcept, Gembloux, Belgium; 4 Kenya Medical Research institute (KEMRI), Nairobi, Kenya; 5 Faculty of Medicine and Soba Teaching Hospital, University of Khartoum, Khartoum, Sudan; 6 Academic Medical Centre, Division of Infectious Diseases, Tropical Medicine and AIDS, Amsterdam, the Netherlands

## Abstract

**Background:**

Molecular methods to detect *Leishmania *parasites are considered specific and sensitive, but often not applied in endemic areas of developing countries due to technical complexity. In the present study isothermal, nucleic acid sequence based amplification (NASBA) was coupled to oligochromatography (OC) to develop a simplified detection method for the diagnosis of leishmaniasis. NASBA-OC, detecting *Leishmania *RNA, was evaluated using clinical samples from visceral leishmaniasis patients from East Africa (n = 30) and cutaneous leishmaniasis from South America (n = 70) and appropriate control samples.

**Results:**

Analytical sensitivity was 10 parasites/ml of spiked blood, and 1 parasite/ml of culture. Diagnostic sensitivity of NASBA-OC was 93.3% (95% CI: 76.5%-98.8%) and specificity was 100% (95% CI: 91.1%-100%) on blood samples, while sensitivity and specificity on skin biopsy samples was 98.6% (95% CI: 91.2%-99.9%) and 100% (95% CI: 46.3%-100%), respectively.

**Conclusion:**

The NASBA-OC format brings implementation of molecular diagnosis of leishmaniasis in resource poor countries one step closer.

## Background

The leishmaniases are a group of diseases with a broad range of clinical manifestations. They are caused by several species of the genus *Leishmania*, in the family Trypanosomatidae. The World Health Organization considers leishmaniasis as one of the most important neglected parasitic diseases [1-3]. Leishmaniasis is endemic in 88 countries with 350 million people at risk of contracting the disease. There is an estimated incidence of 1 to 1.5 million cases of cutaneous leishmaniasis (CL) and 500, 000 cases of visceral leishmaniasis (VL) primarily in South America, East Africa and the Indian Subcontinent.

Early diagnosis of leishmaniasis is important in order to avoid severe clinical manifestations to the patient including mortality for VL patients. Routine diagnosis of leishmaniasis relies on microscopic demonstration of *Leishmania *amastigotes in aspirates from skin, lymph nodes, bone marrow, liver or spleen and on culturing parasites from these sites. However, aspirate sampling is uncomfortable for the patient and the isolation of parasites by culturing is time-consuming, difficult and expensive. A number of indirect serological tests such as enzyme-linked immunosorbent assay (ELISA), rK39 dipsticks [4,5], direct agglutination test (DAT) [6] and the fast agglutination screening test (FAST) [7] have been developed for the sero-diagnosis of VL. Serology for CL or for VL in immunocomprimised individuals may be considered unreliable due to low or absent production of specific antibodies [8]; although some evidence shows successful serological diagnosis of VL in HIV co-infected patients from Ethiopia with the DAT [9].

Sensitive and specific molecular diagnostic tests such as polymerase chain reaction (PCR) have been developed for the diagnosis of VL [10-13] and CL [14,15]. Furthermore, nucleic acid sequence based assay (NASBA), an amplification technique based on RNA detection has been developed for the diagnosis of various infectious organisms including mycobacteria [16], *Plasmodium *[17], *Leishmania *[18] and *Trypanosoma brucei *[19]. Despite high sensitivity and specificity molecular diagnostic tests are at present not ideal for field diagnosis as they employ equipment that is not practical in field conditions and is often expensive. In this study a simple single step dipstick detection technique, Oligochromatography (OC), for the detection of NASBA amplicons of the 18S RNA of *Leishmania *was evaluated. Previously, dipstick technology has been developed in combination with PCR [20] and NASBA [21] for the detection of *T. brucei *in the diagnosis of Human African Trypanosomiasis, leishmaniasis [22,23] and identification of various old world species of *Leishmania *[24]. In the present study the diagnostic performance of a NASBA-OC assay using clinical samples from various clinical presentations of leishmaniasis was evaluated in a typical phase 1 diagnostic study where proof of principle is assessed on clinical samples.

## Results

All tests performed in the current study were valid since there were no test failures observed. In case of an invalid test result the control signal with the artificial Q RNA was absent. A summary of the results is provided in Table 1.

**Table 1:** Summary of the results of NASBA-OC testing of different clinical samples.

Clinical Sample	Number of Samples NASBA-OC positive	Number of Samples NASBA-OC negative
Blood samples healthy controls from Sudan (n = 50)	0	50

Blood samples confirmed VL cases from Sudan (n = 30)	28	2

Skin biopsies of patients with other skin diseases from The Netherlands (n = 5)	0	5

Skin biopsies of patients with confirmed CL from Suriname (n = 27)	27	0

Skin biopsies of patients with confirmed CL from Brazil (n = 43)	42	1

**Sensitivity, specificity, positive (PPV) and negative predictive value (NPV) estimated at a 95% confidence interval (95% CI) of the NASBA-OC on different clinical samples**		

NASBA-OC employed on blood samples		

Sensitivity	93.3% (95% CI: 76.5% - 98.8%)	

Specificity	100% (95 CI: 91.1% - 100%)	

PPV	100% (95% CI: 85.0 - 100%)	

NPV	96.2% (95 CI: 85.7% - 99.3%)	

NASBA-OC employed on skin biopsies		

Sensitivity	98.6% (95% CI: 91.2% - 99.9%)	

Specificity	100% (95% CI: 46.3% - 100%)	

PPV	100% (95% CI: 93.4% - 100%)	

NPV	83.3% (95% CI: 36.5% - 99.1%)	

### Analytical sensitivity and specificity of *Leishmania *NASBA-OC

The analytical sensitivity of the developed *Leishmania *NASBA-OC diagnostic test was assessed using serially diluted parasites from culture and blood spiked with *L. donovani *promastigotes. The NASBA-OC was able to detect 1 parasite per ml (cultured parasites). The species specificity was assessed on non target nucleic acids (*Mycobacterium tuberculosis*, *Plasmodium falciparum*, *Brucella abortus, Salmonella typhi*, *T. b. gambiense*, and *T. b. rhodesiense*) as well as *L. donovani*. The NASBA-OC assay only gave a positive result for nucleic acids from *L. donovani *culture; all other non target pathogens nucleic acids gave negative results.

### Clinical samples

In total, 50 blood samples from healthy controls and 30 blood samples from confirmed VL cases were analysed in the *Leishmania *NASBA-OC test. All healthy control samples were negative with the employed test. Twenty eight of the parasitologically confirmed cases were positive with NASBA - OC. The sensitivity and specificity of the NASBA-OC on blood samples was 93.3% (95% CI: 76.5% - 98.8%) and 100% (95 CI: 91.1% - 100%), respectively. The positive and negative predictive values are 100% (95% CI: 85.0 - 100%) and 96.2% (95 CI: 85.7% - 99.3%), respectively. The strength of agreement between the NASBA-OC for VL and the combined employed parasitological tests is considered to be very good, with an observed agreement of 97.5% (κ value = 0.946; 95% CI: 0.872 to 1.020)

Seventy five skin biopsies were tested of which 70 were from microscopically confirmed CL patients and 5 were from patients with other skin diseases. Of these, 69 samples tested positive for CL, and all the biopsies from non-CL patients tested negative. Only one sample from a CL patient (from Brazil) tested negative. The sensitivity and specificity of the NASBA-OC was 98.6% (95% CI: 91.2% - 99.9%) and 100% (95% CI: 46.3% - 100%), respectively. The NASBA-OC positive and negative predictive values are 100% (95% CI: 93.4% - 100%) and 83.3% (95% CI: 36.5% - 99.1%) respectively for the skin biopsy samples. There was also a very good agreement between microscopy and NASBA-OC with observed agreement = 98.7% of the observations; κ value = 0.902 (95% CI: 0.711 - 1.093) on the skin biopsy samples.

The combined agreement between NASBA-OC for leishmaniasis and the employed diagnostic tests for cutaneous and visceral leishmaniasis is 98.1%, κ value = 0.958 (95% CI: 0.911 - 1.005)

## Discussion

Over the last decade diagnostic tests based on molecular biology techniques have proven to be more sensitive and specific than the classical methods and thus case finding has greatly improved using these techniques [25]; however, implementation of these tools may be difficult for logistical, economic and technical reasons. Among the molecular techniques that have been developed for the diagnosis of leishmaniasis are PCR and NASBA. Here we combine NASBA with Oligochromatographic technology to produce a diagnostic test that may be suitable for implementation in the developing world. The NASBA-OC assay developed in this study is based on the 18S rRNA gene sequence, and this target has been found to be highly efficient for the diagnosis of leishmaniasis from human clinical material [11,13]. The assay can be used to detect any leishmaniasis causing species, since the target sequence is common in all members of genus *Leishmania*; speciation, which maybe important for treatment considerations, can not be made here.

The analytical sensitivity of the NASBA-OC test for leishmaniasis employed in this study, using both the cultured parasites and spiked blood, was 1 and 10 parasites per ml respectively which is slightly more sensitive than that achieved in earlier studies [18,26]. A PCR-OC test for the detection of leishmaniasis reached a sensitivity of approximately 5 parasites/ml blood [23]. In contrast, Laurent et al [24], used the OC dipstick to detect PCR products of *Leishmania *but achieved a 10 times lower sensitivity of 14 parasites per μl of blood. However, this study targeted a different gene, namely the cysteine proteinase B gene.

In the present study, blood samples of parasitologically confirmed VL patients were used for the detection of *Leishmania*. The sensitivity of the NASBA-OC on blood samples from VL patients (>90%) is better compared to PCR studies carried out earlier in Sudan [11] where sensitivity was persistently lower (around 70%). This indicates an advantage of NASBA-OC over conventional PCR, especially in Sudan, where parasitaemia in general is reported to be low [27].

A number of molecular tests have also been developed for the diagnosis of CL. To this end, Bensoussan et al. [14] compared three PCR assays used in the diagnosis of CL; the spliced leader mini-exon PCR had the lowest sensitivity of only, 53.8%, the rRNA gene internal transcribed spacer 1 (ITS1) PCR had 91.0% sensitivity while the kinetoplast DNA (kDNA) PCR achieved the highest sensitivity (98.7%) which is comparable to 98.6% sensitivity achieved in this study for CL. Recently, Deborggraeve et al. [23] developed a PCR-OC based on the 18S rDNA gene for the diagnosis of leishmaniasis and was able to diagnose 91.7% patients with cutaneous leishmaniasis using skin biopsies compared to 98.6% sensitivity found with NASBA-OC in the current study.

Previously, this NASBA amplification assay was used in combination with electro-chemiluminescence (ECL) for the detection of NASBA products. This method is more complex, labour-intensive and requires sophisticated equipment [18]. The sensitivity and the specificity of the NASBA using ECL detection were comparable with the results obtained in this study. Simplification of the detection method, therefore, does not compromise the accuracy of the NASBA as a diagnostic tool. Furthermore, the oligochromatographic dipstick is simpler, faster and more user friendly and better suited for field use than the ECL detection method. Espinosa et al. [22] performed PCR combined with OC in the Peruvian jungle and considered the time reduction achieved a major advantage over conventional diagnostic methods and predict possible implementation of the technology in low-level-equipped laboratories.

A common downside of the technique described in this study is contamination that may arise during the procedure of NASBA, leading to false positive results. Although contamination should be avoided in any other molecular diagnostic tests, the concern is more imperative in NASBA since the test is very sensitive and can amplify such small amounts of nucleic acid compared to other tests, including PCR. To avoid sample contamination the NASBA enzymes that are responsible for amplification are added to the reaction mixture in a special designated room that is considered to be free of amplicons.

## Conclusion

The current paper describes the development of a highly sensitive and specific molecular detection method for both cutaneous and visceral leishmaniasis based on an isothermal amplification method and a simple read-out system. The next step will be to subject the developed assay to rigorous field testing to establish its diagnostic value under resource-poor conditions with clinically suspected patients.

## Methods

### Cultured parasites

Promastigotes of *L. donovani *(MHOM/SD/68/1S) were cultured *in vitro *as previously described [7] and the parasites were reconstituted into 10^6 ^parasites per ml aliquots in phosphate buffered saline (PBS). Ten-fold serial dilutions of the culture were made (10^5^, 10^4^, 10^3^, 10^2^, 10^1^, and 1 parasites per ml) in triplicate. Similarly, the same serial dilutions of parasites were used to seed EDTA-supplemented blood from a non-endemic healthy volunteer with the same parasite numbers.

Nucleic acid from other cultured pathogens, i.e., *Mycobacterium tuberculosis*, *Plasmodium falciparum*, *Brucella abortus, Salmonella typhi*, *T. b. gambiense*, and *T. b. rhodesiense*, were obtained from other research groups and included in this study to assess analytical specificity of the NASBA-OC test

### Clinical samples

Clinical samples obtained from different study locations were used in the present study. All clinical material from Sudan was subject to appropriate ethical clearance from the Faculty of Medicine, University of Khartoum and from the National Ethical Committee at the Federal Ministry of Health Sudan.

Samples from Suriname were collected at the Department of Dermatology, Academic Hospital Paramaribo (Anton de Kom University of Suriname) and at the Dermatological Service, Tourtonnelaan, Paramaribo. The work performed in Suriname was reviewed and approved by the Medical Ethical Committee of the Academic Medical Centre, Amsterdam, The Netherlands (MEC 03/228). Sample collection in Brazil was at Fundação de Medicina Tropical do Amazonas, Manaus, Brazil and approved by the Brazilian National Review Board of the Ministry of Health (Commissão Nacional de Ética em Pesquisa Parecer no. 1142/2005).

Written informed consent was obtained from study cases before clinical samples for research purpose were collected.

#### Clinical samples of visceral leishmaniasis patients

Blood samples (n = 30) from Sudanese visceral leishmaniasis patients, parasitologically confirmed by microscopic examination of bone marrow aspirates, were used in the present study. The samples were processed in the Leishmaniasis Research Laboratory of Soba Teaching Hospital (Khartoum University) and all were found positive for *Leishmania*

#### Clinical samples from cutaneous leishmaniasis patients

Skin biopsies from confirmed CL patients were collected from Brazil (n = 43) and Suriname (n = 27). Parasitological diagnosis was achieved by direct microscopic detection of *Leishmania *amastigotes in Giemsa stained skin smears for patients with CL. A single skin biopsy (2-mm in diameter) was collected under local anesthesia (xylocaine) with a sterile disposable skin biopsy puncher from the active edge of the lesion according to WHO recommendations [3]. Species identification was performed at Royal Tropical Institute, KIT Biomedical Research, Amsterdam, The Netherlands, using standard PCR-RFLP techniques [28]. The samples from Suriname all contained *L. braziliensis guyanensis*; 37 samples from Brazil contained this parasite also, 5 Brazilian samples had *L. b. braziliensis *and one was typed as *L. amazonensis*

#### Endemic controls

Blood was also collected from 50 healthy individuals without clinical signs or complaints associated with leishmaniasis from Sudan to serve as endemic controls. In addition, skin biopsies were collected from individuals (n = 5) with lesions due to other conditions than cutaneous leishmaniasis [18]: ulcerative pyloderma (n = 2), folliculitis (n = 1), dermatitis (n = 1) and sarcoidosis (n = 1). These individuals visited the Dermatology Outpatient clinic of the Academic Medical Centre (Amsterdam, The Netherlands) and are non-endemic patients.

### Extraction of nucleic acid from clinical samples

Skin biopsies (2 mm in diameter) from lesions of CL patients were mixed with 950 μl L6 lysis buffer (50 mM Tris HCl, 5 M GuSCN, 20 mM EDTA, 0,1% Triton- X-100) and stored at -70°C.

Two hundred μl EDTA blood from VL patients was mixed with 1200 μl of L6 lysis buffer and 40 μl of silica and centrifuged. The sediment was kept at -20°C. RNA and DNA were extracted from the samples as described earlier with the Boom method [29].

### NASBA

The NASBA employed in this study targets a 170-bp region in the 18S rRNA, and was performed using the Nuclisense BasicKit (BioMérieux) for amplification following the procedures published previously [18]. The primers and probes employed in the amplification are presented in the Appendix. The NASBA reaction comprised three enzymes needed for replication; i.e. AMV-RT, RNase H and T7 RNA polymerase, which are added to the reaction mixture in an amplicon free room to reduce the change of sample contamination, and was performed in a 10 μl total reaction volume at a final KCl concentration of 70 mM containing 0.416 μM of each primer

Artificial constructed *in vitro *RNA, referred to as Q RNA, (10^6 ^molecules per reaction) derived via site-directed mutagenesis, was added to the NASBA mix and serves as an internal amplification control (Appendix). A sample containing water was used as a negative control.

The reaction mixture was incubated in a 0.5 ml tube with 2.5 μl RNA extract at 65°C for 2 minutes and then at 41°C for 2 minutes. The NASBA enzymes were subsequently added and isothermal amplification took place for 90 minutes at 41°C in a heat block. All samples were tested 3 times for reproducibility. Amplified RNA product was detected using the oligochromatography detection method.

### Detection by oligochromatography

Oligochromatographic (OC) detection of NASBA products (for more details on the OC test see: http://www.corisbio.com/public/product/documents/ProductFileLeishmania.pdf was completed in an assay tube preheated at 55°C. 4 μl of NASBA amplification product was mixed with 76 μl of migration buffer, preheated at 55°C, in an assay tube and subsequently an OC dipstick was dipped into the mixture. The assay tube was sealed with a cap and incubated at 55°C for 10 minutes, after which both sides of the OC dipsticks were read [20-24].

### Statistical analysis

The sensitivity and specificity of the *Leishmania *NASBA-OC test were calculated using the formulas: TP×100/(TP+FN) and TN×100/(TN+FP) respectively, where TP, TN, FP and FN represent; true positives, true negatives, false positives and false negatives. The positive and negative predictive values were calculated by analysis of 2 × 2 contingency tables. All calculations were estimated at a 95% confidence interval (95% CI).

Furthermore, the degree of agreement between the evaluated tests was determined by calculating Kappa (κ) values with 95% confidence intervals using Epi-info version 6. Kappa values express the agreement between tests that is beyond chance; a κ value of 0.60 to 0.80 represents a substantial agreement beyond chance, whereas a κ value of > 0.80 represents almost perfect agreement beyond chance.

## Competing interests

The authors declare that they have no competing interests, except for the following. TL is an employee of Coris BioConcept (Gembloux, Belgium), the company that is producing OC-based tests for PCR. However, this affiliation did not limit the execution of the work nor did it interfere with the interpretation of test results or the conclusion of the research.

## Authors' contributions

CM: Study design, molecular work, statistical analysis drafting manuscript. TL: OC-test development, commenting on manuscript. GS: Molecular work, pathogen cultures, drafting of manuscript. FB: Molecular work, pathogen cultures, commenting on manuscript. AS: Collection of clinical samples, molecular work. SS: Collection of clinical samples, commenting on manuscript. PK: Study design, collection of clinical samples, commenting on manuscript. HS: Study design, statistical analysis, commenting on manuscript, corresponding author.

## Appendix

Primers and probe sequences employed in the *Leishmania *18S NASBA assay and oligochromatography

18S Leishmania primers sequences

Leish 18S F GEN^1^.5'-**GATGCAAGGTCGCATATGAG**CCAAAGTGTGGAGATCGAAG -3'

Leish 18S T7 R^2^5'-*AATTCTAATACGACTCACTATAGGGAGAAG*GGCCGGTAAAGGCCGAATAG -3'

18S Leishmania probe sequences

Capture probe NASBA Leish Wt: 5'-AGACCATTGTAGTCCACACTGC-3' -biotin

Capture probe NASBA Leish Q: 5'-CTTAGGTCCACTAAGGTACC-3' -biotin

Detection probe NASBA Leish: 5'- GATGCAAGGTCGCATATGAG-3'

Leish-QRNA:

5'CCAAAGTGTGGAGATCGAAGATGATTAG***CTTAGGTCCACTAAGGTACC***CAAACGATGACACCCATGAAT

TGGGGATCTTATGGGCCGGCCTGCGGCAGGGTTTACCCTGTGTCCAGCA

CCGCGCCCGCTTTTACCANCTTACGTA TCCTTTCTATTCGGCCTTTACCGGCC 3'

^1^The sequence in bold is a generic tail enabling the hybridization of the NASBA product by the detection probe.

^2^The region which is presented in italics is the T7 promoter sequence.
